# Multi-subject/daily-life activity EMG-based control of mechanical hands

**DOI:** 10.1186/1743-0003-6-41

**Published:** 2009-11-17

**Authors:** Claudio Castellini, Angelo Emanuele Fiorilla, Giulio Sandini

**Affiliations:** 1 DIST, University of Genova, viale F Causa 13, 16145 Genova, Italy; 2 Italian Institute of Technology, via Morego 30, 16163 Genova, Italy

## Abstract

**Background:**

Forearm surface electromyography (EMG) has been in use since the Sixties to feed-forward control active hand prostheses in a more and more refined way. Recent research shows that it can be used to control even a dexterous polyarticulate hand prosthesis such as Touch Bionics's i-LIMB, as well as a multifingered, multi-degree-of-freedom mechanical hand such as the DLR II. In this paper we extend previous work and investigate the robustness of such fine control possibilities, in two ways: firstly, we conduct an analysis on data obtained from 10 healthy subjects, trying to assess the general applicability of the technique; secondly, we compare the baseline controlled condition (arm relaxed and still on a table) with a "Daily-Life Activity" (DLA) condition in which subjects walk, raise their hands and arms, sit down and stand up, etc., as an experimental proxy of what a patient is supposed to do in real life. We also propose a cross-subject model analysis, i.e., training a model on a subject and testing it on another one. The use of pre-trained models could be useful in shortening the time required by the subject/patient to become proficient in using the hand.

**Results:**

A standard machine learning technique was able to achieve a real-time grip posture classification rate of about 97% in the baseline condition and 95% in the DLA condition; and an average correlation to the target of about 0.93 (0.90) while reconstructing the required force. Cross-subject analysis is encouraging although not definitive in its present state.

**Conclusion:**

Performance figures obtained here are in the same order of magnitude of those obtained in previous work about healthy subjects in controlled conditions and/or amputees, which lets us claim that this technique can be used by reasonably any subject, and in DLA situations. Use of previously trained models is not fully assessed here, but more recent work indicates it is a promising way ahead.

## Background

Electromyography (EMG from now on) is a well-known diagnostic tool for detecting muscle disorders from motor unit activation potentials [1,2]. In its non-invasive (surface) version it has also been used since the Sixties [3-5] to enable amputees control one or two degrees-of-freedom (DOFs) of active upper limb prostheses. Its commercial/clinical applications include, e.g., Otto Bock's SensorHand Speed [6], the Motion Control Hand and the Utah Arm [7], and more recently, Touch Bionics's i-LIMB [8], with 5 active and one passive DOF. In some of these cases, force/torque are also controlled.

The popularity of surface EMG stems from its cheapness, simplicity of use and non-invasiveness.

Nevertheless, research on more and more dexterous mechanical hands is ongoing (e.g., the DLR-II hand [9] and the Cyberhand [10,11]) and soon a finer control will be required. To this end, at least since 2002 [12-15] it is known that a few surface EMG electrodes suffice to recognise up to nine isometric/isotonic hand postures. This potentiality has so far been exploited clinically in the i-LIMB only, and to a very limited extent so far, as far as we know. In previous work it has also been shown that a dexterous hand prosthesis can be feed-forward force-controlled *while *detecting grasping postures [15,16] in real time. So it appears that plain, old EMG still has to be exploited in full.

The work presented in this paper fits in this line of research, extending previous results along two "orthogonal" directions: first, we analyse data collected from 10 healthy subjects and thus try and assess the general applicability of the technique; second, we compare a baseline controlled condition with a "Daily-Life Activity" (DLA) one, in which subjects walk, raise their hands and arms, sit down and stand up, etc., while performing the same actions of the baseline. The DLA condition is an experimental proxy of what a patient is supposed to do in real life. Lastly, we propose a cross-subject model analysis, i.e., training a model on a subject and testing it on another one. The use of pre-trained models could be useful in shortening the time required by the subject/patient to become proficient in using the prosthesis.

## Materials and methods

### Subjects

Ten healthy subjects joined the experiment after having given their informed consent. The subjects were two women and eight men, nine right-handed and one left-handed, average age 30.9 ± 8.45 years, standard Caucasian weight and height. They were given no knowledge of what the experiment was about.

### Experimental procedure

**Figure 1:**
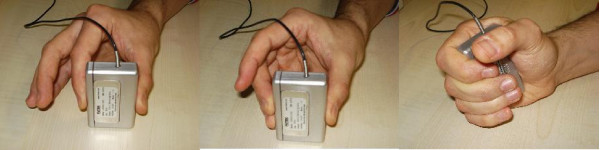
**The three different grips employed in the experiment: (*left*) index precision grip; (*center*) other fingers precision grip; (*right*) power grasp**.

The experiment consisted of two phases. During phase 1, after a "rest" condition was sampled to define the baseline EMG activity, the subject would keep her/his arm still and relaxed on a table, and was asked to grasp a force sensor using, in turn, three different ways of grasping it (Figure 1): index precision grip, other fingers precision grip and power grasp. While gathering data, a label {1, 2, 3, 4} denoting the grasp (or rest) was attached to each sample, in order to build the ground truth values.

The subject freely repeated each grasping action for 100", resting for 30" in between grasps. The whole procedure was repeated twice for numerical robustness purposes. This "baseline" phase will be referred to from now on as the *Still-Arm phase (SA)*.

Phase 2, which started soon after phase 1 for each subject, consisted in repeating phase 1 while the subject was left free to move, walk around, lift and pronate/supinate the arm and forearm, sit down and stand up from a chair. This second phase is intended as a laboratory-controlled proxy of the main movements a patient is expected to do during DLAs. This phase will be called *Free-Arm phase (FA)*.

Each subject's experiment resulted in something more than 1200" of data. Data were sampled at 2 KHz, resulting in about 2.4 × 10^6 ^samples for each subject, equally distributed in each phase.

### Equipment and electrode placement

**Figure 2:**
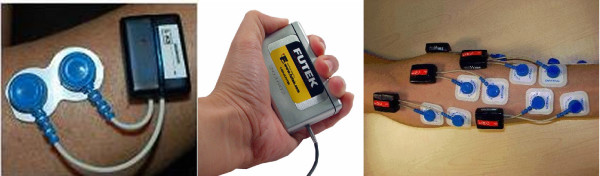
**Part of the experimental setup: (*left*) an EMG wireless electrode; (*center*) the force sensor; (*right*) typical placement of the EMG electrodes on a subject's forearm (ventral side)**.

We employed Aurion ZeroWire wireless surface EMG electrodes [17], in order to ease the FA phase, which required free movement in the laboratory. A FUTEK LMD500 Hand Gripper force sensor [18] was used to detect the force applied while grasping. (See Figure 2.) A standard digital acquisition board (National Instruments NI-USB6211) was used to record the signals, connected to the receiver of the EMG wireless device and to an amplifier, in turn connected to the force sensor. The sampling rate was set at 2 KHz in order to correctly sample both signals (the EMG signal relevant bandwidth lies between 15 and 500 Hz). The board was connected via a USB port to an entry-level laptop. We used a custom National Instruments' LabView VI block to acquire the signals.

Seven electrodes were glued on each subject's dominant forearm, according to this anatomic guideline:

• on the forearm ventral side: near the wrist, above the *flexor pollicis longus*; centrally, above the *flexor digitorum superficialis*; near the elbow, above the *flexor digitorum profundus*; and near the wrist, above the *flexor digitorum superficialis *again;

• on the forearm dorsal side: near the wrist, above the *extensor pollicis brevis/abductor pollicis longus*; centrally, above the *extensor digitorum communis *and *extensor digiti minimi*.

These positions were chosen, according to the medical [19] and bioengineering [20] literature, to detect the activity of the flexor and extensor muscles of the forearm which are most relevant during grasping. Figure 2 (rightmost panel) shows the typical electrode positioning. Notice that there may be remarkable inter-arm differences depending on the subjects' age, gender and physical fitness. Moreover, some of the aforementioned muscles are deep into the forearm, so that muscle cross-talk cannot be avoided. This is a well-known problem in the EMG literature [1,13].

### Data analysis

The root-mean-square (RMS) of the EMG was evaluated using a time window *T*_*RMS*_. The optimal value of *T*_*RMS *_was evaluated independently for classification of the grasping posture and force detection, via grid search, in a preliminary phase of the experiment, and set to 500 *ms *for classification and 100 *ms *for regression. The choice of the RMS, as opposed to the simpler rectification and filtering, is motivated by its well-known relationship to the force exerted by the related muscle [1,2,13]. Rectification plus filtering would likely work as well, and it is indeed employed in some commercial myoelectrodes such as Otto Bock's MyoBock ones [21].

Notice that the right choice of *T*_*RMS *_can be, in general, crucial: a small value will make the system more responsive (i.e., implies a smaller delay) but a higher value will be more informative and improve the performance (especially in the case of classification, as we verified). On the other hand, it is known that the EMG signal anticipates the muscle movements by a few hundreds milliseconds; therefore, in a practical application derived from this experiment, a wider lag would be more acceptable than one would expect. The electromechanical delay (EMD) of a muscle is defined as the interval between the onset of the electrical activity of the muscle (EMG) indicating its activation by the neural system and the onset of the resulting change in the mechanical variable observed. The delays reported range from 25 to 100 *ms *for different muscles and tasks [22].

**Figure 3:**
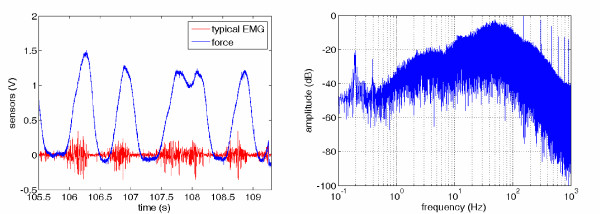
**(*left*) Typical raw EMG (red) and force (blue) signals, as read from the electrodes and force sensor; (right) frequency diagram of the EMG signal**.

**Figure 4:**
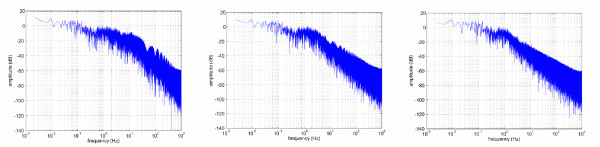
**(*left to right*) Effects of the RMS on the bandwidth of the EMG signals, for *T*_*RMS *_= 20, 100, 500 *ms***.

Figure 3 (left) shows the typical EMG signal (red) and force (blue) recorded by the force sensor. Clearly the amplitude of the envelope of the EMG is related to the force, as is indicated in literature. The right panel of the Figure shows the bandwidth of the EMG. Figure 4 shows the effect of the RMS on the frequency components of the EMG, for three different values of *T*_*RMS*_. In all cases, the RMS signal bandwidth is upper-bounded by about 25 Hz (left panel, for *T*_*RMS *_= 20 *ms*) to 10 Hz (right panel, for *T*_*RMS *_= 0.5*s*), as expected (larger values of *T*_*RMS *_correspond to a better filtering but also to a larger delay). According to these figures, we subsampled the RMS of the EMG signals at 25 Hz by taking one sample in 80 of the original sequence, resulting in about 30.000 samples for each subject.

Lastly, samples for which the applied force was lower than a specific threshold were removed. After verifying several choices both numerically and visually, the threshold was uniformly set at 20% of the mean force value obtained for each subject and phase.

### Statistical analysis

According to previous literature (e.g., [14,16]), the statistical analysis was carried on using Support Vector Machine (SVM). For a comprehensive tutorial on SVMs refer to [23,24]. SVMs are a statistical learning method able to build an approximated map between an input space and a label (classification) or a real value (regression). Classification is here used to classify the type of grasp according to the EMG signal, whereas regression is used to understand how much force the subject is exerting, independently from the grasp type. The input space is ℝ^7^, one coordinate for each EMG electrode. We used the ground truth values as labels and the force value given by the force sensor for the regression. Notice that SVMs work here in real-time, associating a grasp type and a force value to an EMG value at each instant of time. Grasp type and forces are then predicted almost at the onset of the grasping movement, differently from what happens in other approaches (e.g., [14,25]) in which all values of the input signal over a further time-window are employed as the input space.

In order to ease the computational burden we employed uniformisation [16] to reduce the size of the training sets. The samples in a training set are considered one by one in chronological order, as it would happen in an on-line setting, and each new sample is added to the training set if and only if its Euclidean distance from all training samples retained so far is larger than a predefined value *d*. Values of *d *were set to 0.02 for the SA phase and 0.032 for the FA phase. These values were chosen in order to get not more than one thousand training samples for subject 1. The choice is arbitrary, but notice that (see [16] again) the performance of such systems changes linearly as *d *changes, whereas the training set size varies polynomially; thus, it is always possible to find a polynomially smaller training set, if needed, which will degrade the performance only linearly. This really means that the initial choice of *d *is not crucial. Also, notice that testing sets have not been uniformised, in order to give a more realistic result.

SVM analysis was performed for each subject and for each phase, to check how the performance depends upon subjects and conditions. For classification, the performance index is, as is customary, the percentage of overall correctly guessed labels. For regression, the performance index is the correlation coefficient evaluated between the predicted force signal and the real one. The choice of the correlation coefficient is suggested by this consideration: when driving a prosthesis we are not interested in the absolute force values desired by the user/subject, since mechanical hands usually cannot apply as much force as human hands do, for obvious safety reasons (or, e.g., in teleoperation scenarios, they could be able to apply *much more *force than a human hand can). Rather, we are concerned about getting a signal which is strongly correlated with the user/subject's will. Anyway, we also report about the normalised root mean-square error (NRMSE), in order to give a broader view of the results. Normalisation is done against the signals' ranges (notice, though, that correlation is the criterion used to find the optimal parameters during grid search). We employed a well-known freely available SVM package, *libsvm *v2.83 [26], in the Matlab wrapped flavour; the Gaussian kernel was chosen, since it is a standard choice in previous literature. EMG data were normalised along each dimension, as is customary, by subtracting the mean value and dividing by the standard deviation. 5-fold cross-validation was used to assess the generalisation error for each training set; this measure was then used for grid-searching the typical Gaussian kernel hyperparameters of a SVM, called *γ *and *C*. Once these parameters were found, the overall performance was evaluated as the mean and standard deviation of the performances obtained on each fold.

## Results

### Per-subject analysis

**Figure 5:**
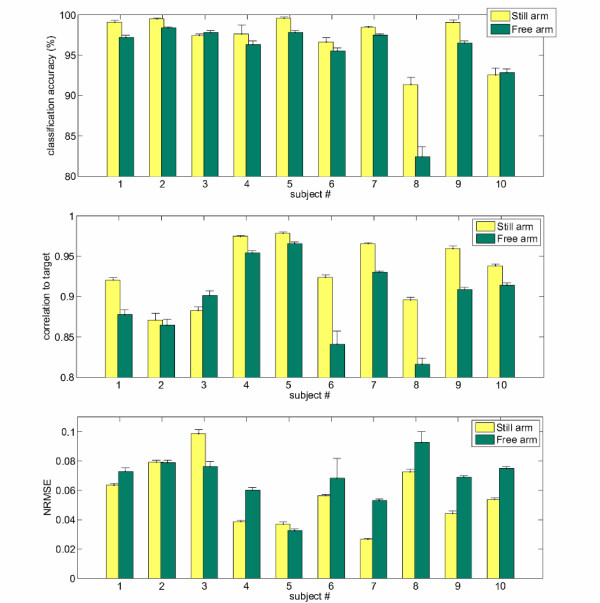
**Classification (*top*) and regression (*middle*, correlation to target; *bottom*, NRMSE) results obtained by the system, on both phases of the experiment (FA and SA) and for each subject**.

Figure 5 shows the main results. Classification accuracy (top panel) for the SA phase ranges from 99.58% ± 0.17% (subject 5) to 91.37% ± 0.89% (subject 8); for the FA phase, it ranges from 98.40% ± 0.08% (subject 2) to 82.43% ± 1.24% (subject 8 again). On average over all subjects, the classification accuracy is 97.14% ± 2.90% for SA and 95.24% ± 4.77% for FA. Notice that the performance is consistent by subject and by phase, meaning that (*a*) hard subjects in the SA phase are hard as well in the FA phase and viceversa, and (*b*) the FA phase is always harder than the SA phase.

Regression figures (middle and bottom panels) show that for the SA phase the correlation to true signal ranges from 0.9784 ± 0.0017 (subject 5) to 0.8959 ± 0.0033 (subject 8), whereas for the FA phase it ranges from 0.9657 ± 0.0022 (subject 5) to 0.8161 ± 0.0078 (subject 8). On average, the correlation is 0.93 ± 0.04 for the SA phase and 0.90 ± 0.05 for the FA phase. Again, consistency by subject and by phase appears. Remarkably, not all subjects which are slightly harder for regression (namely, 1, 2, 3, 6, 8) happen to be hard for classification; in particular, only subject 8 is definitely hard *both *for classification and regression, while, e.g., subject 6 is hard for regression but not that hard for classification. The bottom panel shows that an analogous situation appears if we consider the NRMSE. (Recall that the NRMSE is an error measure while the correlation to target is a positive performance index.)

**Figure 6:**
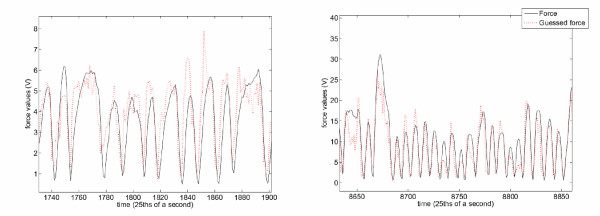
**Comparing true (black continuous line) and guessed (red dotted line) force values for regression of a typical subject (number 6, FA phase)**.

**Figure 7:**
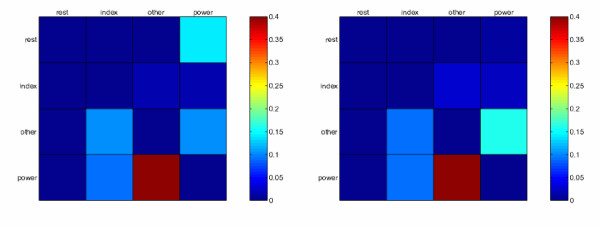
**Confusion matrices for the SA (*left*) and FA phase (*right*)**. Each matrix is the average over the confusion matrices of the 10 subjects. A confusion matrix *C *is such that its (*i*, *j*)th element is the fraction of *i *labels mistaken for *j *labels, over the total mistaken labels.

Figure 6 shows the real and guessed force values for a typical subject, namely number 6, FA phase. Strong correlation between the guessed and true values is visually apparent, in agreement with the performance values outlined before. On the other hand, Figure 7 shows the (average) confusion matrices for the SA and FA phases. Clearly, most of the classification errors, for both phases, regard the "power grasp" being mistaken for the "other fingers precision grip". This is intuitively sensible, since gripping with middle, ring and pinkie finger involves co-contracting the index finger too, to some extent. This makes the former grip quite similar to the latter, from a muscular point of view.

As far as hyperparameters grid search is concerned, Table 1 shows the average values of (the logarithms of) *γ *and *C *for the optimal models obtained via cross-validation. The grid search ranges were [0, 3] for *log*_10_(*C*) and [-1.85, 0.16] for *log*_10_(*γ*) (these are standard values in literature, given the dimensionality of the input space). The average value of *log*_10_(*C*) is around 1.5, but its standard deviation is rather wide with respect to the range, at least in the case of classification. The standard deviation is smaller for regression than for classification in both cases, which seems to indicate that regression is more stable a problem with respect to the hyperparameters.

**Table 1:** Mean values and standard deviations of the hyperparameters *γ *and *C*.

Phase, problem	***Log***_**10**_**(*γ*)**	***log***_**10**_**(*C*)**
SA, class.	-0.35 ± 0.58	1.6 ± 0.84
FA, class.	-0.65 ± 0.54	1.55 ± 0.83
SA, regr.	-0.50 ± 0.24	1.45 ± 0.44
FA, regr.	-0.60 ± 0.26	1.45 ± 0.37

**Figure 8:**
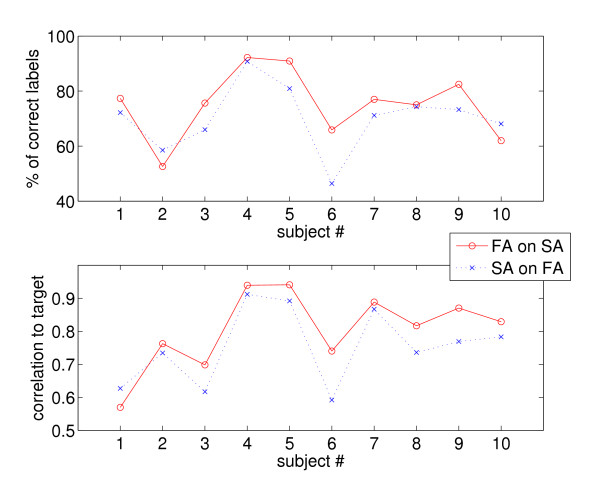
**Classification (*top*) and regression (*bottom*, correlation to target) results obtained testing on SA-data models trained on FA, and vice-versa**.

In order to check whether the FA phase is really indicative of what a patient might do in her/his DLAs, we have trained a machine on the data gathered during the FA phase and then tested it on the data gathered during the SA phase. Figure 8 shows the results of testing FA-models on SA data, and viceversa.

FA-models tested on SA data obtain an average accuracy of 75.11% ± 12.34% for classification and 0.8056 ± 0.1151 for regression; whereas testing SA-models on FA data gives 70.17% ± 11.99% in classification and 0.7530 ± 0.1153 in regression. The advantage of FA models over SA models is apparent, uniform and consistent. Notice that here we show no error bars, since, for each subject and phase, there is just one training set and one testing set.

**Figure 9:**
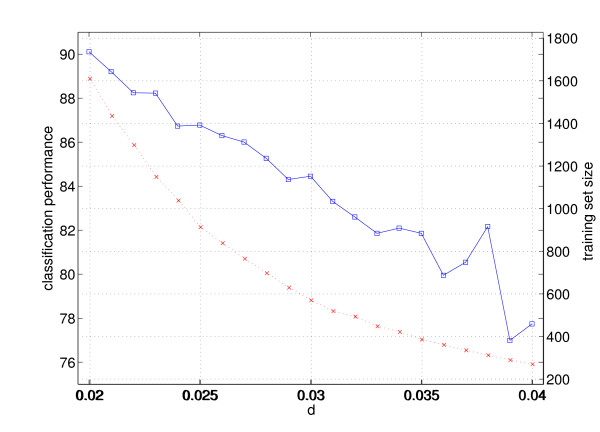
**Size of the training set (red dotted line) and classification performance (blue continuous line), of subject 8 in the FA phase, as *d *changes**.

Lastly, let us consider the worst result of the per-subject analysis -- subject 8 in the FA phase, as far as classification is concerned. One of the possible causes of this comparatively low performance (82.43% ± 1.24) is that too many samples are missing from the original training set (*d *too high). In order to test this hypothesis, we let *d *linearly range around the pre-set value of 0.032 and check (*a*) the size of the resulting training set and (*b*) the performance obtained by the system. Figure 9 shows the result of this test.

The Figure confirms that the training set size has a decreasing polynomial trend, while the performance changes linearly [16]. In particular, for *d *= 0.032 the previously shown performance appears, whereas if a larger performance is required, one can increase the number of samples in the training set, or, which is equivalent, reduce the magnitude of *d*. For instance, to get an accuracy of about 90% *d *must be set at 0.2 ending up in a training set with some 1600 samples.

### Cross-subject analysis

**Figure 10:**
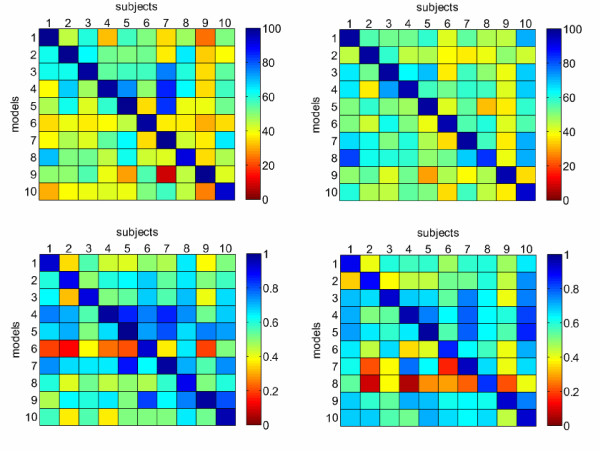
**Cross-subject performance matrices, for classification (*top row*) and regression (*bottom row*), in the SA (*left column*) and FA phase (*right column*); the numbers refer to all element of the matrices, excluding the diagonals**.

Recall that in this experiment, for all subjects, the EMG electrodes were carefully positioned on the forearm according to an anatomical guideline, meaning that noise due to inter-arm differences should be to some extent avoided. We can therefore check how well each model performs on each subject by building a cross-subject performance matrix A, for both classification and regression, and for both phases, in which *A*_*ij *_is the performance index attained by a model trained on data gathered from subject *i *while predicting data gathered from subject *j*. Figure 10 shows the matrices.

The overall results indicate that a large amount of the models overlap, or at least that there is a certain cross-subject capacity of prediction. Consider the numbers below the matrices in the Figure: in classification, the performances are 51.69% and 54.04%, with the remarkable particular that the FA-models are slightly, but consistently, *better *in cross-subject analysis (higher mean values and lower standard deviations) than the SA-models. As far as regression is concerned, the average cross-subject correlation is around 0.60. Notice that models trained on subjects 6 (for the SA phase) and 8 (FA phase) appear to be particularly bad in predicting other subjects' data (the related rows of the bottom left and right matrices, in turn, are rather darker than the average).

In previous work it was shown that a significant (inverse) correlation appears between the cross-subject performance matrices and the *cross-distance matrices D*, obtained by evaluating a mean distance *D*_*ij *_between two sample sets *S*_*i *_and *S*_*j *_like this:

This analysis for each pair (*i, j*) of subjects and for the two phases and problems shows that inverse correlation is absent in the case of the FA phase in classification; it is mild (-0.32) for SA in classification; and that it is strong in the case of regression (-0.63 for the SA phase and -0.65 for the FA phase). It is likely that the correlation in regression is connected to the actual smoothness of the function the system is trying to approximate. It is unclear why the classification problems show a weak correlation or none at all.

## Discussion and conclusion

Since 2002 at least, it is known that that machine learning methods, applied to EMG-based hand/wrist configuration recognition, can solve the problem quite thoroughly (an incomplete list includes [11,16,27-29]). The research is all the more interesting since very recent work on amputees, both from the neuroscientific [30,31] and the engineer's [32-34] point of view, clearly shows that it is applicable to the disabled. Within this stream of research, this work aims at answering two questions:

1. can this technique be applied to any (healthy) subject?

2. will it work in Daily-Life Activities?

The results presented above point at a positive answer to both questions.

The first question is answered by noting that a uniformly good performance is obtained for each subject, in each phase. The figures obtained by on the SA phase are comparable to those found in other, related work such as [11,34] or [16] where the predicted signals were actually used to control the DLR-II hand in real-time. This indicates that the approach will reasonably work on any healthy subject. Combining this result with the more recent results obtained on amputees listed above, one can conclude that the approach is viable for a wide range of patients, too. Notice that SVMs are by no means the only approach to solve this problem; linear regression, neural networks, LWPR [35] and Hidden Markov Models [27], among others, have been employed too, with similarly good results; probably, even simpler approaches would get an acceptable level of performance, which further raises the hopes for a real system based upon these results. From the point of view of machine learning, interpreting surface EMG is an easy task, a feeling corroborated, at least in the case of regression, by the uniformity of the optimal hyperparamters found by grid search.

The second question is here equivalent to asking whether the performance is comparable between the SA and FA phases, provided that the FA phase is a reasonable experimental proxy of DLAs of the standard patient. The results obtained in the FA phase are actually in the same order of performance as those in the SA phase. A deeper analysis reveals that FA models are in a sense "wider" than SA ones, since they test better on SA data than the reverse.

As an aside result, it turns out that uniformisation produces small training sets (about 30 times smaller than the original, subsampled sets) which are used to generate models with excellent accuracy. The phenomenon described in [16] is here confirmed: as the minimum distance *d *is linearly increased, performance degrades linearly while the training sets become polynomially smaller. This opens up the possibility of using it to build asymptotically bound training sets, which is paramount in an on-line setting, where the data flow is potentially endless.

Notice that, in this work, the training sets are, in absolute terms, *small*, since each subject could not be tested for more than 20 minutes; this means that the models presented here might suffer from noise introduced by medium-to-long term factors such as, e.g., muscle fatigue, sweat and/or electrode re-positioning. In [16] it is shown that these problems could be overcome by a sufficiently long training time, and we see no reason to believe that this is not the case here.

Also notice that, in general, predicting the grip force from the EMG signal is nothing new -- the EMG-to-force is well-known and has been modelled, among other methods, via linear regression [36]. Our regression model is novel in that it predicts the force to a similar degree of precision independently of the grasp type employed. So it can be used in parallel with the classifier, as it has indeed been done in [16]. As far as cross-subject analysis is confirmed, the figures presented here cannot be used in practice, although they are better than chance; but notice that in [37] a more refined approach has been employed successfully, indicating that pre-trained models can be effectively used to improve classification and regression performance, with respect to *tabula rasa *learning.

## Competing interests

The authors declare that they have no competing interests.

## Authors' contributions

CC has collected some data, performed the data analysis and written most of the paper. AEF has taken care of the setup, collected most of the data and written some of the paper. GS has helped design the experiment, proof-read the paper and given useful advice throughout the realisation of the work. All authors have read and approved the manuscript.
